# Aptamer-Gated Nanoparticles for Smart Drug Delivery

**DOI:** 10.3390/ph4081137

**Published:** 2011-08-15

**Authors:** Veli Cengiz Ozalp, Fusun Eyidogan, Huseyin Avni Oktem

**Affiliations:** 1 University of the Basque Country, Polymat, Avda. de Tolosa, 72, Donostia-San Sebastian 20018, Spain; 2 Nanobiz Ltd. Co., METU Technopolis, Gallium Block, 2nd floor, No.18, Ankara 06531, Turkey; 3 Faculty of Education, Baskent University, Ankara 06810, Turkey; 4 Department of Biology, Middle East Technical University, Ankara 06531, Turkey

**Keywords:** aptamers, nanoparticles, drug delivery, molecular gating, nanovalves

## Abstract

Aptamers are functional nucleic acid sequences which can bind specific targets. An artificial combinatorial methodology can identify aptamer sequences for any target molecule, from ions to whole cells. Drug delivery systems seek to increase efficacy and reduce side-effects by concentrating the therapeutic agents at specific disease sites in the body. This is generally achieved by specific targeting of inactivated drug molecules. Aptamers which can bind to various cancer cell types selectively and with high affinity have been exploited in a variety of drug delivery systems for therapeutic purposes. Recent progress in selection of cell-specific aptamers has provided new opportunities in targeted drug delivery. Especially functionalization of nanoparticles with such aptamers has drawn major attention in the biosensor and biomedical areas. Moreover, nucleic acids are recognized as an attractive building materials in nanomachines because of their unique molecular recognition properties and structural features. A active controlled delivery of drugs once targeted to a disease site is a major research challenge. Stimuli-responsive gating is one way of achieving controlled release of nanoparticle cargoes. Recent reports incorporate the structural properties of aptamers in controlled release systems of drug delivering nanoparticles. In this review, the strategies for using functional nucleic acids in creating smart drug delivery devices will be explained. The main focus will be on aptamer-incorporated nanoparticle systems for drug delivery purposes in order to assess the future potential of aptamers in the therapeutic area. Special emphasis will be given to the very recent progress in controlled drug release based on molecular gating achieved with aptamers.

## 1. Introduction

Nanoparticles are increasingly becoming of interest in medical applications for drug delivery, metabolite sensing, and diagnostics. They are among the various innovative approaches in reformulating new delivery systems for optimized delivery of drugs. The classical approach to ensure a required therapeutic concentration is administration of drug at high and repeated amounts when the distribution is non-selective. Other than inconvenience to patient the major draw-back with this approach is directly correlated with the negative side-effects of the administered drug. Nanoparticle-encapsulated drug delivery aims to deliver the active therapeutic ingredients to the disease site in stable compartments in order to reduce premature release. This ensures that the effects of drug are maximized and the side effects are reduced. An encapsulated nanoparticle system requires a specific targeting mechanism and at the same time the retention of drugs inside the container should be high. The balance between specificity of targeting and the extent of premature leakage determines the success of a given delivery system. Nanotechnology research approaches in drug delivery include a wide variety of nanomaterials ranging from soft hydrogels to solid polymeric particles. Large surface area, high drug loading efficiency and potential combination with other organic/inorganic materials are the main properties of hollow nanostructures that are attractive for biomedical applications. Packaging of small-molecule drugs improves their availability, compatibility and reduces toxicity. To achieve these advantages, nanoparticles should incorporate mechanisms for efficient targeting and controlled release.

Controlling the drug release profile is the main challenge in drug delivery development when the drug is to be successfully targeted to a specific site. Stimuli-responsive materials have been created by using biological, physical and chemical properties of materials for heat-activated, light-activated or pH-activated delivery. Special formulations like albumin-based, carbohydrate-enhanced, or fatty acid targeting have also been utilized. In other approaches, nanoparticles systems were capped with materials that respond to stimuli such as temperature, pH, light, electromagnetic waves, oxidation potential, enzymatic activity or other biological markers. Nucleic acids are utilized to construct rationally designed nanostructures at molecular levels for nanotechnology applications. Integration of the properties of nucleic acids can offer many opportunities for drug delivery systems, including stimuli-responsive nanogates for nanocarriers and molecular sensors [1]. Favorable drug release kinetics can be achieved at the target sites by aptamer-based capping systems. Nucleic acid functionalization for targeting or sensing purposes has been exploited for decades using a variety of materials such as aluminium, silica, or carbon nanotubes [2].

## 2. Aptamers

Nucleic acid aptamers are single stranded short oligonucleotide sequences with high affinity to specific targets [3]. In Nature, they exist as a nucleic acid based regulatory elements called a riboswitch. Aptamers are powerful targeting agents for nanoparticles because it is relatively straightforward to immobilize them on nanosurfaces without changing the affinity properties. They are widely considered as nucleic acid antibodies with additional superior properties. Artificial selection, *in vitro* chemical synthesis, economic production, relatively easy modification, and physical stability are the superior properties of aptamers over antibodies. *In vitro* selection of aptamers is a major advantage over other biorecognition agents. Systemic Evolution of Ligands by EXponential enrichment (SELEX) is a type of combinatorial chemistry methodology that is designed to identify aptamer sequences [4,5]. It is a relatively fast and completely *in vitro* established procedure. The manual nature of the procedure was later converted into an automated method for high-throughput production [6]. However, the number of aptamers available has always been limited. A recent report by Gold *et al.* [7] reported multiplex DNA-aptamer based array for 813 proteins. This study can be considered as an indication for future potential on the accessibility of aptamers.

Several inherent characteristics make aptamers excellent active binding elements. First of all, artificial selection is a powerful technique in producing aptamers for any kind of target and moreover to a specific region of the target. Aptamers are physically stable, which means they can survive extreme conditions like high temperature [8] and extreme pH values [9]. Automated versions of the selection procedures have been successful to some extent, providing high-throughput selection of new aptamers. On the other hand, there are aptamer properties that should be treated with caution. The application of aptamers in biological environments requires the consideration of nucleases ubiquitously found in blood and intracellular environments. In this respect, DNA is more resistant to nuclease attack in applications involving blood, and RNA is more resistant inside the cells. Various approaches to stabilize aptamers has been explored and most success obtained by spielmegers (L-enantiomers of nucleic acids) [10] and secondary modifications (e.g., 2′-amino-, 2′-fluoro-) [11-13]. The modifications usually should be applied *a priori* to the SELEX procedure rather than *a posteriori*. A promising approach in obtaining cell specific aptamers is a recent adaptation of traditional selection methodology. Cell-SELEX identifies aptamers by using whole cells as target molecules during selection procedure. The availability of cell-specific aptamers is essential in developing drug targeting systems since pathology modifies normal cell to have marker properties which can easily be used for selecting aptamers [14].

## 3. Nanoparticles for Targeted Pharmacotherapy

Nanoparticles have been accepted to be one of the best media for developing target drug delivery. Diverse nanostructure forms have been proposed for drug delivery, including liposomes, polymeric particles, microemulsions, hydrogels, or polymeric micelles. They are termed with different names such as nanocarriers, nanocapsules, nanospheres, and hollow materials. However, drug encapsulation has been achieved via two main approaches: (i) homogenous matrix systems in which drugs are dispersed, absorbed, dissolved, covalently attached; or (ii) encapsulation systems in which drug solutions are confined inside a shell-like wall. The encapsulation of drugs in nanocompartments is usually capped with stimuli-responsive elements for targeted and controlled delivery applications. Potentially any material that contains inside spaces and is smaller than one micron can be used in developing a new drug delivering nanoparticle system. There are several criteria in choosing the nanoparticle material dictated by the final application. Nanoparticles in therapeutic applications need to be biocompatible, biodegradable, and non-toxic. In the body, the cargo of nanoparticles is usually released by diffusion, swelling, erosion or degradation. The major advantages of nanoparticles in drug delivery are large overall surface area, high loading capacity, and high solid content dispersions at low viscosity. Targeted polymeric nanoparticles have been demonstrated to benefit the therapy by delivering higher total fractions of drugs to target cells [15], enhancing intracellular drug delivery through receptor mediated endocytosis and reducing unintentional leakage of drugs [16].

**Figure 1:**
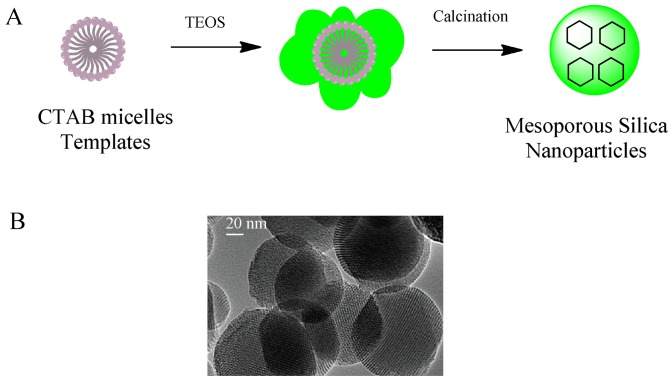
(**A**) Mesoporous silica nanoparticles were typically synthesized at three steps; (i) an organic template micelles of cetyltrimethylammonium bromide (CTAB) were created; (ii) then tetraethylorthosilicate (TEOS) was added to form a silica network around the micelles (depicted in green); and (iii) the organic template was removed by heating (calcinations); (**B**) Transmission electron microscopy (TEM) image of mesoporous silica particles show honeycomb-like structures [19] [TEM image is reproduced with permission, Copyright John Wiley and Sons].

Mesoporous silica nanoparticles have been excellent candidates that fulfill many of the requirements for targeted and controlled drug delivery since they have been proposed as a drug delivery system [17]. They are well-defined nanostructures synthesized through a sol-gel procedure (Figure 1).

**Figure 2:**
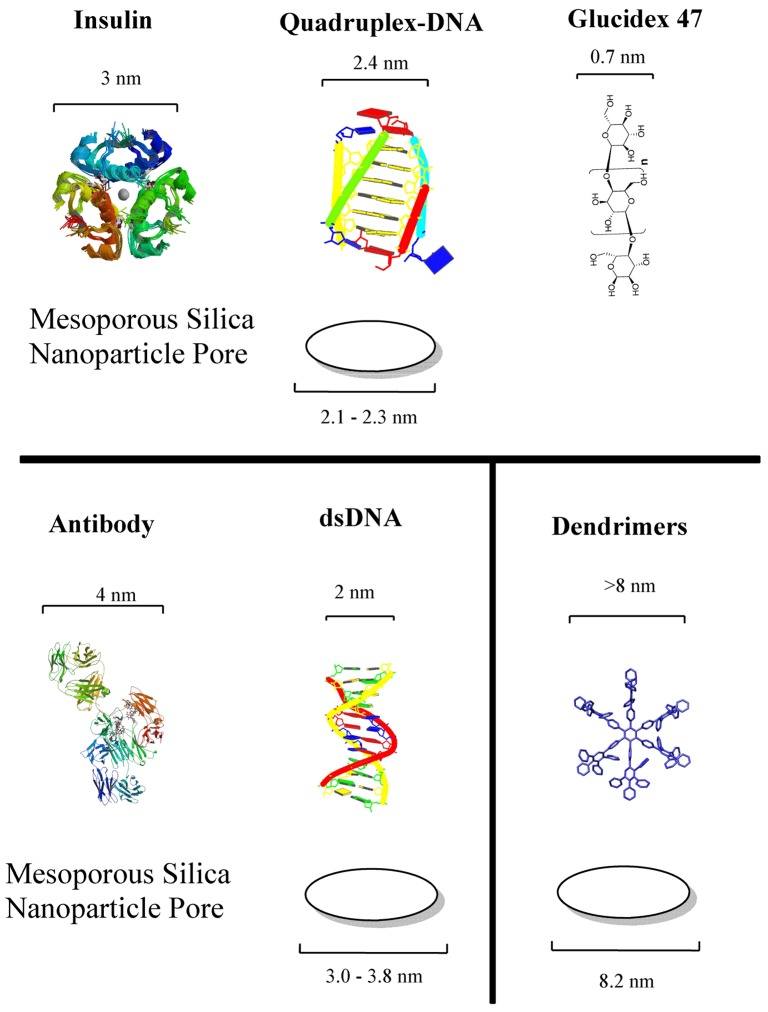
The dimensions of soft caps and corresponding silica pore sizes as reported in literature. Various mesoporous pore diameters have been used to achieve successful capping function with each biomolecule [26,28,34-36]. The dimensions of capping molecules are approximate and derived from RSCB protein databank by using software Jmol for insulin (PDB ID:1AI0), antibody (PDB ID:1IGT), i-motif DNA (PDB ID:1CN0) and double stranded DNA (dsDNA, PDB ID:1BNA). The dimensions for Glucidex 47 is based on the size of glucose molecules (KEGG ID: C00031) and dendrimer size was from Arotiba *et al.* [37].

Drug loading properties and easy immobilization of biomolecules on the surface can be mentioned as the two main advantages. They are known for their ordered structures and high loading capacity. Mesoporous silica possess present convenient properties for the development of drug delivery systems. In fact, the majority of stimulus-responsive nanocomposites involve mesoporous silica nanoparticles. Detailed review articles devoted entirely to mesoporous silica or the drug delivery applications of mesoporous silica can be found elsewhere [18,19]. Different drug molecules have been shown to be trapped inside nanoporous materials, capped for stable delivery and release through a trigger mechanism. The internal cavities of nanoporous materials provide safe microenvironments for drugs that can be loaded and protected from degradation during transport. The stability of drug-loaded mesoporous particles is usually achieved through blocking the pore mouths, termed as “capping”. There exist two kinds of capping material with properties designed for targeting or stimuli-responsive releasing requirements. Hard caps are materials such as iron oxide [20], cadmium sulfide [21] or gold nanoparticles [22]. Soft caps include organic molecules, biomolecules and supramolecular structures. The cap material is usually selected at a size that can match the pore size since the main purpose of the cap is to prevent the leaching of drug as much as possible. Biomolecule-based caps were developed for specialized task and enzymes [23,24], sugars [25,26], antibodies [27] and nucleic acids [28] are among the types of molecules. Figure 2 shows the dimensions of examples of biomolecules used for capping the corresponding pore sizes of mesoporous silica.

The most common proof-of-concept molecules used as drug models are fluorescent molecules since loading and release of cargo molecules can readily be monitored through fluorescence techniques. Water soluble fluorescein or rhodamine or their common derivatives are the fluorescent molecules preferred by researchers. Both molecules are reasonably similar to common drug molecules in size and general chemical properties. Therefore, it is assumed that the final developed drug delivery system could be optimized by using fluorescent molecules and that they can be used by replacing them with real drug molecules in real therapeutic applications in the future. Doxorubicin is the preferred drug for model systems when they are tested on *in vitro* systems. Doxorubicin is a chemotherapeutic agent used in the treatment of some types of cancer, mostly leukemia and neuroblastoma. It is an anthracycline molecule with antibiotic and antitumor activities that slows down the growth of cancer cells. Its mode of action is through intercalating into the minor groove of DNA inducing a local unwinding in the helix structure. Doxorubicin was encapsulated in liposomes [29] for treatment of Kaposi's sarcoma [30], multiple myeloma [31], breast [32] and ovarian cancers [33].

## 4. Aptamer-Based Targeting for Nanoparticles

Selective delivery of drugs has always been a desired product in achieving maximum therapeutic effects on target areas of body. Nanoparticle-based delivery systems have to be tailored with consideration of the target in order to improve the therapeutic effects of the drug. There have been many achievements in drug delivery for specific applications, but challenges remain for bottom-up approaches to design targeting tools that can generically be applied to any drug delivery task. Biorecognition elements such as antibodies or aptamers have been successfully incorporated into nanoparticles for targeting. Aptamer selection methods have traditionally used purified target molecules, which is very similar to antibody production methods. The SELEX procedure is a combinatorial identification methodology based on an efficient affinity selection technique. The cell-specific markers are mostly membrane-bound molecules and the purified samples do not completely represent the molecular tertiary structure in membranes. The early selection approaches tried to overcome this difficulty by over-expressing marker proteins on recombinant cells and using the whole cell in the SELEX procedures (called cell-SELEX). Cell-SELEX is an extension of the traditional SELEX procedure by using whole cells in the aptamer evolution procedure. Therefore, aptamer selection relies on the efficiency of the specific separation of affinity targets. This approach resulted in a limited number of cell-specific aptamers which have been exclusively used in targeting applications to date, namely tenacin-C, PSMA, gp120, nucleolin, transferrin, mucin-1 protein tyrosine kinase 7, immunoglobulin heavy Mu chain, epidermal growth factor receptor, prostate tumor cells, HIV-infected cells [30]. Aptamers specific for leukemia [38], lung cancer [39], colorectal cancer [40], ovarian cancer cells [41] have been selected by using cell-SELEX methodology. However, the success of aptamer-based targeting in real therapy will depend on the availability of aptamers for any desired application. The recent advances in cell-SELEX procedures promise to expand the availability of cell-specific aptamers. The whole cell is a complex target, meaning that affinity-separation cycle in SELEX is a challenge and novel approaches are needed. For example, next-generation sequencing together with new bioinformatic data mining techniques can provide strategies to overcome the complexity in identification of aptamers for whole cell targets. In fact, recent reports indicate potential progress in future applications. For example, Bayrac *et al.* [42] recently employed pyrosequencing for selecting aptamers for glioblastoma tissue, which is the most common brain tumor in humans. Potentially, the new cell-SELEX techniques should be expected to result in more cell-specific aptamers for incorporation in targeted drug delivery systems. Such novel approaches are essential for the future of aptamer-based systems because widespread availability of aptamers is the main advantage over antibodies. The recent literature reported new cell-specific aptamers for new kind of potential drug delivery targets. It should be expected increasing number of studies for drug delivery systems based on aptamers targeting more specific cell types. Table 1 is a list of cell-specific aptamers commonly used in targeting of nanoparticles and Table 2 presents the main examples of aptamer-based medical applications. The targeting application involving nanoparticles will be briefly summarized for each aptamer, focusing on individual examples. Detailed reviews on aptamers in targeting can be found elsewhere [43,44].

### 4.1. Prostate Cancer Targeting Aptamer

Prostate specific membrane antigen (PSMA) aptamer has been a model aptamer in aptamer-based targeting research activities since it was selected in 2002 [45]. PSMA aptamer, also called A10 aptamer, is an RNA sequence and thus usually 2′ fluoro-modified and has an inverted T cap at the 3′end of molecule to increase its stability when used in targeting studies. PSMA is a type II integral membrane glycoprotein found in prostate tissue and overexpressed in prostate cancer cells. It can be used as a prostate cancer marker. PSMA aptamer was successfully used for drug targeting and bioimaging. Dose escalated therapy is one type of beneficial cure for prostate cancer and damage to non-cancer tissue is the major problem in this therapy. Sensitizing agents improve the therapeutic effect, but an efficient targeting should be employed. DNA-activated protein kinase peptide (DNAPK) was determined as an excellent sensitizing agent [46]. In an effort to design a therapeutic system, shRNA was delivered to PSMA positive cells by PSMA-RNA aptamers in order to selectively reduce the amount of DNAPK in human prostate tissue [47]. Another attractive characteristic of PSMA proteins are that they are continuously recycled through endocytosis, making them attractive model systems for intracellular delivery of nanoparticles.

In another therapeutic application, A10 RNA aptamer was immobilized on the surface of thermally cross-linked supermagnetic iron oxide nanoparticles conjugated through streptavidin to doxorubicin and targeted to PSMA positive cells [53]. PLGA-b-PEG (poly(D,L-lactic-co-glycolic acid)-β-(poly ethylene glycol) nanoparticles are loaded with cisplatin and surface functionalized with A10 aptamers [54]. The targeted delivery of cisplatin using this NP system has been shown to improve tolerability an efficacy in a xenograft mouse model. Gold nanoparticles functionalized with PSMA aptamers was demonstrated to target to PC3 prostate epithelial cells which express PSMA more than surrounding other kind of cells [55].

### 4.2. Leukemia Targeting Aptamer

Leukemia is uncontrolled proliferation of blood cells that most of the time leads to the death of patients. Acute lymphoblastic leukemia ranks among the most diagnosed malignancies of children. Protein tyrosine kinase-7 is a marker protein for common human cancers, including colon cancer, lung cancer, gastric cancer and leukemia although the reason for upregulation is not yet clear. In an effort to target leukemia cancer tissue, daunorubicin-loaded single-walled carbon nanotubes were functionalized with sgc8c aptamers and targeted to leukemia cells [63]. The aptamer sgc8c was selected for acute lymphoblastic leukemia T-cells. Another selection was conducted for cell lineages HL60 and NB4. The aptamers specifically differentiating between NB4 and HL60 cell line which are closely related to each other resulted in an example showing the capabilities of the cell-SELEX procedure. Chen *et al.* [64] attached leukemia specific aptamers, sgc8c and TD05 to fluorescent nanoparticles for monitoring the corresponding cell lineages. Another similar monitoring application with silica nanoparticles was reported by Medley *et al.* [65].

### 4.3. Nucleolin Targeting Aptamer

Nucleolin is a cellular membrane protein highly expressed in cancer cells. It is a multifunctional protein and the most representative function is antiproliferative action that blocks the antiapoptotic pathway. The aptamer AS1411 sequence was discovered during unrelated experiments investigating triplex-forming oligonucleotide [52,66]. It is a unique aptamer that was not selected but discovered by chance. Quantum dots nanoparticles conjugated to AS1411 aptamer is reported for targeting HeLa cells [67]. A cobalt ferrite nanoparticle system has been demonstrated to target cancer cells in mouse bearing C6 tumor cells for imaging purposes [68].

### 4.4. Other Targeting Aptamers

There are other cell-specific aptamers which are not yet used for drug delivery, but there exist nanoparticle-conjugates potentially useful in drug targeting. Small-cell lung cancer (SCLC) cell specific aptamers were conjugated with magnetic nanoparticles and used to extract them from mixed cell media. Lung cancer is one of the leading causes of cancer mortality and SCLC is a subtype with the highest rate of dissemination tendency. Recently another SCLC binding aptamer selection was reported for a different lineage of cells [69]. Epidermal Growth Factor Receptor (EGFR) is involved in many types of cancer. An RNA aptamer, J18 was selected and attached to gold nanoparticles [50]. The gold nanoparticle-aptamer nanocomposites were shown to be targeting epidermoid carcinoma cells rather than EGFR-free breast cancer cells. EGFR is known to be internalized and gold nanoparticles were demonstrated to enter into the cells after binding on the surface of cells. The human epithelial carcinoma culture cell line A431 cells express EGFR and the anti-EGFR aptamer was used to identify that cell type [70]. A DNA aptamer for the extracellular domain of the mouse transferring receptor was selected in order to deliver a lysosomal enzyme into mouse fibroblasts to correct the defective glycosaminoglycan degradation [51]. Transferrin is a receptor transporting iron into cells through endocytosis and ubiquitous in mammalian cells. Along with PSMA and EGFR aptamers, transferring aptamer is another target which can deliver the nanoparticles inside the cells through aptamer-based targeting.

### 4.5. Cytotoxicity of Aptamer-Nanoparticle Conjugates

The choice of nanomaterial in developing nanoparticles for drug delivery is a challenge because significant considerations must be given to the human and environmental effects of the nanomaterials. The main cellular responses to nanoparticles include anti-oxidative response, proinflammatory effects, lysosome peameation, mitochondrial membrane potential decrease, caspase activation, cell apoptosis, and cell death [71]. Gold nanoparticles and carbon-based nanoparticles are widely reported for their cytotoxic effects on cells [72,73]. For example, single-walled carbon nanotubes and 10 nm silica particles coated with albumin have been reported to induce anti-inflammatory responses in macrophages [74]. Mesoporous silica did not pose any *in vitro* cytotoxicity at low concentrations or *in vivo* cytotoxicity in some studies [75], but they cause cell damage at higher concentrations [76]. However, potential toxicity of nanoparticles in therapeutic applications should still be a concern. Nanoparticles interact with cells first through their surfaces which are commonly modified by the adsorption of other molecules. The biological impacts of nanoparticles are affected by the nature of materials used in surface modification [77]. The surface properties, charges, aggregation and size of nanoparticles are important considerations affecting the interactions with cells. The drastic affect of the type of surface modification on toxicity has been shown with mesoporous silica nanoparticles [78,79]. Significantly reduced rates of heamolysis have been achieved by polyethylene glycol (PEG) coating of silica nanoparticles [80]. Long term cytotoxicity tests are needed to confirm the clinical safety [81].

The toxicological properties of aptamers are not well reported, although detailed information exists for antisense oligonucleotides and other oligonucleotides [82], so at least similar effects to those seen with oligonucleotides should be expected with aptamers. Unspecific interaction of oligonucleotides with proteins, typically observed at high concentrations, can affect protein function. Activation of alternative pathways (complement activation) results in pseudohypersensitivity responses [83]. Complement activation has been reported to result in death in monkeys through cardiovascular syndrome characterized by hypertension and tachycardia [84,85]. Another reported toxic effect of oligonucleotides is anticoagulation. Prolonged coagulation times were observed through low-affinity interactions with the tenase complex [86]. The accumulation of oliginucleotides in certain tissues or cells was observed by immunochemistry and histopathology [87].

## 5. Stimulus Responsive Controlled Release with Nucleic Acids

One of the main objectives of targeted delivery is to achieve controlled release once the drug is at a specific location in the body. For nanoporous capsule-type nanoparticles, on-demand release can be performed through molecular gating. Selective permeation is one of the major phenomena in transport across biological membranes. For example, transmembrane proteins can transport ions in response to a variety of stimuli, including light, oxidoreduction, electrochemical potential, phosphate bond hydrolysis [88]. The stimuli-responsive nanostructured systems have recently attracted significant attention in the research of drug delivery. Numerous surface-functionalized mesoporous nanoparticles have been designed to achieve minimum premature-release through efficient capping systems. Among them, pH-responsive nanoparticles have been promising systems for cancer drug delivery over conventional carriers. Nanomaterials have been shown to efficiently release drugs selectively at cancer cells when physical properties of nanoparticle delivery system respond to pH changes. Several pH-sensitive nanocontainer systems have been developed by using: (i) liposomes [89]); (ii) conformation changing polymers; or (iii) nanoparticles capped with acid-labile molecules [90]. However, the notion of using nucleic acids for gating purposes in drug delivery is quite recent. In the following sections, the early examples in which nucleic acids or aptamers that have been used as stimuli-responsive gates for time-releasing mechanism that control the rate and period of drug delivery will be summarized.

### 5.1. Electromechanical Applications

Nucleic acid functionalization of porous materials provided sensing of the ion flux in order to use it for a variety of nanobiotechnological applications. An electrical rectifier was created by coating the walls of 30 nm gold nanotube pores embedded in polymeric membranes [91]. In another study, pH-sensitive quadruplex was immobilized at the nanopores in order to control ion flux [92]. I-motif oligonucleotides undergo conformational changes in response to pH between four strand densely-packed structure at low pH and random single-strand structure at higher pH values. A sensor type gate was constructed with protein-DNA hybrids [93].

### 5.2. Nucleic Acid-Based pH-Responsive Gating

The simplest way of nucleotide capping of pores of nanocontainers is the adsorption of single stranded DNA oligonucleotides. Climent *et al.* [94] modified mesoporous silica nanoparticles with amino groups to create a positively charged surface which interacts with negatively charged DNA molecules. Thus, the interaction of single stranded DNA molecules with the surface attached positively charged amino groups resulted in an effective capping of the pores. The nanoparticle cargo can be released when complementary DNA strand binds and displaces the adsorbed oligos on the surface of nanoparticles. Although complementary DNA triggered release does not present many applications, this was an early example proving that nucleic acids can be effectively used in capping of mesoporous particles. Later pH-sensitive release was achieved when the single stranded DNA was chosen to have quadruplex structure [28]. The structure of quadruplex forming oligonucleotides is sensitive to pH. A pH-triggered assembly of single-walled carbon nanotube was created by functionalizing carbon nanotubes with complementary DNA oligonucleotides at neutral pH values [95]. However, acidic environments cause the DNA sequences to stabilize in G-quadruplex or i-motif structure by melting hybridized sequences and leading to disassembly of whole structure. The switching property of DNA functionalized single-walled carbon nanotube assembly/disassembly was designed to produce swelling-shrinking material which is proposed as a pH-dependent delivery system for drugs.

### 5.3. Nucleic Acid-Based Thermo-Responsive Gating

Capping of nanoporous particles can be achieved by covalently attaching a double stranded helix of oligonucleotides near the pore openings [28,36]. Recently, thermo-responsive nanocontainers were created by using this strategy [96]. First a single stranded oligonucleotide was immobilized on the surface of silica nanoparticles. The capping mechanism was prepared by attaching a complementary DNA sequence to magnetic nanoparticles. The magnetic nanoparticles close the pores of silica nanoparticles when the complementary DNA sequences hybridize to each other at low temperatures. The melting temperature of the hybridized sequence was adjusted to 47 °C, which corresponds to the upper limit of therapeutic hyperthermia. Thus, the cargo would be released when hyperthermia in the body approaches to upper limit by releasing the therapeutic content. In fact, this type of design can be used to develop capping systems which can be released at any predetermined temperature. In an earlier study, a similar temperature-triggered system was developed by Schlossbauer *et al.* [36]. The pores of mesoporous silica MCM-41 with 3.8 nm pore opening was capped first by avidin and then by double stranded DNA labeled with biotin at one end. The authors reported two different length caps with 15 and 25 nucleotides which are opening the pores at 45 °C and 65 °C, correspondingly.

**Figure 3:**
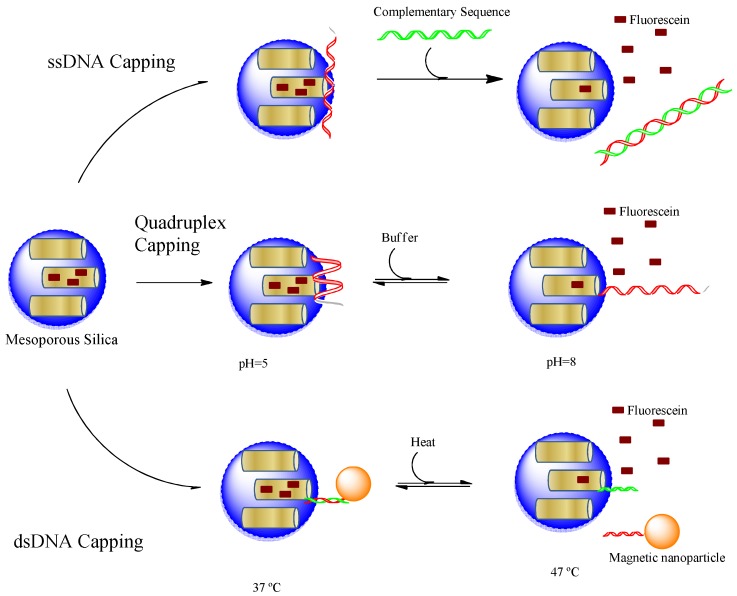
DNA oligonucleotide capping mechanisms; single stranded, quadruplex or double stranded DNA were used to cap mesoporous silica nanoparticles.

Figure 3 summarizes all three approaches using oligonucleotides as capping material, which indicates that the structural variety of nucleic acids can provide stimuli-responsiveness for different kinds of triggers. Single stranded oligonucleotides were used to create DNA responsive on-demand cargo release while quadruplex structures were designed to respond to pH changes. The DNA hybridization mediated capping could result in temperature responsive devices. Furthermore, aptamers can be used to construct materials responsive to any kind of biological molecule from small metabolite to whole cells as will be discussed in the next section.

## 6. Aptamer-Based Gate Keeping on Mesoporous Silica Nanoparticles

Combined with mesoporous nanoparticles stimuli-responsive capping has been used to create smart delivery systems. The stable, rigid frame of silica allows for transport of drugs encapsulated inside the particles resistant to pH, biological degradation or mechanical stress. The interaction of drugs with environment is prevented through an efficient capping mechanism until the particles arrive to the specific locations throughout the body. The diameters of pores of mesoporous silica particles can be tuned between 1 to 10 nanometer scale. The mechanical motions of nanovalves such as rotaxanes or azobenzenes can precisely be controlled at the molecular scale and their dimensions are comparable in size to nanopores of mesoporous particles [97-99]. In addition to such physical and chemical stimuli, biological triggering should be exploited for an improved development of drug delivery to pathology-related markers. Enzymes and antibodies were the first biomolecules to be considered. The pores of mesoporous material were previously capped by antibody for controlled release applications [94]. Although the gating was controlled through an antibody-antigen interaction, the gating mechanism was quite specific to sulfothionine antibodies. The control of pore opening was based on competition of antibody on heparin and sulfothionine. Potentially antibody capping would deliver cargo of nanoparticles to any target by selecting specific antibodies. However, designing of a general opening mechanism is still a challenge. Enzyme-responsive nanoparticles could be created through caps labile to degradation by specific enzymes. Saccharide derivatives were used to cap mesoporous nanoparticle pores and pancreatin or β-D-galactosidase have been shown to digest the caps and release the cargo [26]. In a similar application, mesoporous particles were capped through a porcine liver esterase labile ester-linked adamantyl cap [23]. Both enzyme-responsive and antibody-mediated capping are based on specific interactions with a specific biological stimulus. A new capping system has to be designed for each new application. The linkers for caps should be selected as a point of attack by the specific enzyme or as a competitive binder for the antibody. Unfortunately, such linkers are not common and thus finding an antibody or enzyme responsive linker is a considerable challenge. In that respect, aptamers are suggested as unique materials which can provide biorecognition and a generic opening mechanism upon interaction with trigger. Aptamers have very similar properties to antibodies in achieving high affinity biorecognition, but with additional favorable properties of nucleic acids that is a structure prone to switching. The structural changes can be precisely designed to open or close the nanopores with a trigger of biorecognition. The major advantage of the system is that molecular switching designs apply to any aptamer sequence without changing their affinity properties once the systems was designed to work so.

Aptamer-controlled release of nanoparticle cargo can be considered as a natural extension of the above-mentioned nucleic acid-based systems with additional properties. The aptamers are single-stranded nucleic acids with a function attributed by their tertiary structures. Therefore, aptamers can be manipulated in the same way as any nucleic acid oligonucleotides. This makes adaptation of the same systems for nucleic acids applied directly to aptamer-based systems. However, the aptamer gating will provide a release mechanism triggered by aptamer-target interaction. Therefore, aptamer-based systems should be considered as a mechanism that can extend the stimuli for any kind of target in the cell environment. The feasibility of aptamer use as specific ligands has been proven in numerous examples in drug targeting, sensing and other nanotechnological areas. There are two very recent reports using aptamers as the gating mechanism in a snap-top system [53] or in a nanovalve-type gating [55].

### 6.1. Aptamer-Based Snap-Top Gating

**Figure 4:**
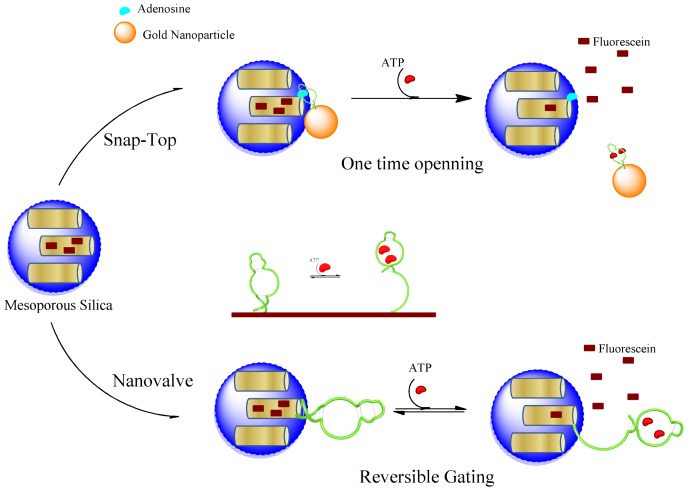
Schematic illustration of aptamer-target responsive controlled drug delivery systems. Two approaches has been adapted by using aptamers as molecular gates; (**a**) Snap-top gating; and (**b**) nanovalves.

A novel stimulus-responsive drug delivery system was recently developed by using aptamer-target interactions [100]. Mesoporous silica nanoparticles were capped with gold (Au) nanoparticles which are linked to the surface of silica through an aptamer sequence binding its target immobilized on the surface of nanoparticles. By competitive displacement, Au particles were uncapped in the presence of trigger molecule ATP and the guest molecules are released (Figure 4). In this work, the solid MCM-41 nanoparticles were functionalized at the-NH_2_ group and a derivative of the ATP molecule (adenosine-50-carboxylic acid, denoted as adenosine-COOH) was immobilized on the outer surface of the MS through an amidation reaction. Meanwhile, Au nanoparticles (AuNPs) were functionalized with ATP aptamer through Au-S bonds. ATP aptamer recognizes the adenine and ribose moieties, when mixing AuNPs-aptamer with adenosine modified MCM particles. AuNPs would be capped on the pores of nanoparticles, owing to the binding reaction of ATP aptamer with adenosine. The release of the guest molecule could be triggered by challenge with ATP molecules. The addition of ATP resulted in a competitive displacement reaction. Therefore, the developed aptamer-based delivery system encapsulates cargo molecules, and delivers the content when encounters an environment with ATP molecules.

### 6.2. Aptamer-Based Nanovalves

The early example of aptamer-based nanovalves was reported for regulating ion transport. A 20 and 65 nm glass nanopores was modified with an aptamer sequence, which undergoes conformational changes in response to its target, cocaine [101]. The glass nanopore electrode was a platinum microdisc embedded at the bottom of a conical nanopore made in glass. Redox-active molecules diffuse through the pore and are monitored electrochemically. The cocaine-binding aptamer was attached in the cavities of pore, and it is partially unfolded in the absence of cocaine. In the presence of cocaine molecules, the aptamers form a three-way junction structure with interaction with the target. The three-way junction occupies less space compared to an unfolded structure, and allows more ions to pass through the pore. Thus, a cocaine-induced gating function was created by reducing the resistance to ion transport in the presence of cocaine. A single synthetic nanopore has been created and shown to mimic the biological pores as gated ion channels. Recently Ozalp *et al.* [102] adapted a similar approach to controlled release of mesoporous silica cargo. A switchable nanovalve using directly an ATP-binding aptamer sequence (Figure 4) was covalently attached on the surface of nanoparticles. The drug delivery system was centred on the conversion of an aptamer sequence into a molecular beacon type hairpin structure, which was used as a nanovalve. The hairpin of the aptamer sequences had originally been designed for signalling the presence of target molecules. This system was found to be highly reversible, which means that partial delivery of drug molecules can be controlled better instead of a sudden release as is the case in snap-top designs.

## 7. Future Perspectives on Combined Drug Targeting and Release

Future drug delivery research should benefit from further developing and improving aptamer-based systems, to include development of nanoparticle systems targeting real world situations. Current aptamer-based research efforts in drug delivery focused on targeting of therapeutic molecules with many successful examples of biological applications on cell culture or mouse models proving the potential of aptamer-based targeting. The research on aptamer-based gating is still at the very early phase without any reports on biological model systems. However, the premise offered by aptamer-based triggers is potentially attractive and future interest in this area should be expected. The encouraging results obtained by aptamer-based gating should generate huge potentials for future thereutic applications in drug delivery field. The ability to deliver drug molecules in response to specific stimuli-molecules makes aptamer-based drug delivery systems a unique therapeutic agent for spatially and temporally controlled drug release. The future applications of individual therapy medicine should benefit greatly from precise delivery and release tools based on aptamer gating.

## Figures and Tables

**Table 1:** Commonly used aptamer sequences used in targeting nanoparticles.

**Name**	**Sequence of Aptamer**
Leukemia Cell Lines (Acute Human Lymphomas T-Cells)	
**Sgc8c**	ATC TAA CTG CTG CGC CGC CGG GAA AAT ACT GTA CGG TTA GA
**KH1C12**	ATCCAGAGTGACGCAGCATGCCCTAGTTACTACTACTCTTTTTAGCAAAC, specific for T-Cells [48]
**TD05**	AAC ACC GGG AGG ATA GTT CGG TGG CTG TTC AGG GTC TCC TCC CGG TG, specific for Ramos Cells [49]
Prostate Specific Membrane Antigen	
**A10**	GGGAGGACGAUGCGGAUCAGCCAUGUUUACGUCACUCCUUGUCAAUCCUCAUCGGC [45]
Epidermal Growth Factor Receptor	
**J10**	GGCGCUCCGACCUUAGUCUCUGCAAGAUAAACCGUGCUAUUGACCACCCUCAACACACU UAUUUAAUGUAUUGAACGGACCUACGAACCGUGUAGCACAGCAGA [50]
Tranferrin receptor	
**FB4**	TGAGGGCGGAAGAACTAATTTGGGACGGATTGCGGCCGTTGTCTGTGGC, extracellular domain of mouse transferring [51]
Nucleolin	
**AS1411**	TTGGTGGTGGTGGTTGTGGTGGTGGTGG [52]

**Table 2:** Examples of medical applications with aptamers.

**Type of Application**	**Aptamer**	**Remarks**
Drug Delivery	PSMA	Targeting of nanoparticles to tumour cells shown in mice [54,56,57]
Bioimaging	AS1411	Colocalization of proteins in HeLa cells [58]
Diagnostics	Somamers	An assay for 813 protein was applied to clinical study of chronic kidney disease [7]
Gene Therapy	CD30	Anaplastic lymphoma kinase gene was silenced by siRNAs in cell culture [59]
Tissue Engineering	Endothelial Progenitor Cells	Capture of endothelial cells on synthetic devices [60]
Pathogen Detection	*Streptococcus pyogenes*	Multiple M-types of Streptococcus bacteria was differentially detected [61]
Metabolite Sensing	ATP	Spatiotemporal ATP profiles in yeast cells [62]
